# Evaluation of the diagnostic efficiency of fluorescence *in situ* hybridization for pulmonary tuberculosis: a systematic review and meta-analysis

**DOI:** 10.3389/fmed.2024.1467530

**Published:** 2025-01-06

**Authors:** Yu-Qi Hu, Kang Liu, Le-Qing Lai, Yi-Ru He, Li-Ping Hong, Chu-Qian Jiang, Si-Min Liu, Ming-Zhu Cao

**Affiliations:** ^1^Department of Obstetrics and Gynecology, Center for Reproductive Medicine, Guangdong Provincial Key Laboratory of Major Obstetric Diseases, Guangdong Provincial Clinical Research Center for Obstetrics and Gynecology, Guangdong-Hong Kong-Macao Greater Bay Area Higher Education Joint Laboratory of Maternal-Fetal Medicine, The Third Affiliated Hospital of Guangzhou Medical University, Guangzhou, China; ^2^Department of Clinical Medicine, The Third Clinical School of Guangzhou Medical University, Guangzhou, China; ^3^Department of Clinical Medicine, The Second Clinical School of Guangzhou Medical University, Guangzhou, China

**Keywords:** fluorescence *in situ* hybridization (FISH), pulmonary tuberculosis, diagnostic accuracy, meta-analysis, probes (sensors)

## Abstract

**Objective:**

In clinical practice, an accurate and efficient detection approach for pulmonary tuberculosis (PTB) is highly needed. The fluorescence *in situ* hybridization (FISH) assay for PTB might be a suitable alternative to current tests. However, a systematic assessment of the diagnostic performance of this new approach is not available. Our study aimed to determine the diagnostic accuracy of FISH for PTB.

**Methods:**

We examined PubMed and three more databases including Embase, Cochrane Library, and Web of Science databases from their establishment to November 10, 2023, for published articles on the diagnostic performance of FISH on individuals with clinical suspicion of tuberculosis (TB). QUADAS-2 was used to evaluate the literature’s quality. We used Meta-DiSc software to create forest plots.

**Results:**

The search yielded 7 studies, involving 1,224 sputum samples that could be included in our meta-analysis. The combined FISH sensitivity and specificity were 0.89 (95% CI 0.86–0.92) and 0.98 (95% CI 0.97–0.99), respectively. Furthermore, subgroup analysis was performed based on probes and PTB incidence.

**Conclusion:**

FISH may be useful in the diagnosis of pulmonary tuberculosis. The sensitivity and specificity of FISH are high for most sputum specimens. Additionally, FISH has better diagnostic performance in countries with low PTB prevalence than in high PTB prevalence countries. We hope this study will find a new and effective tool for the early diagnosis of PTB.

## Background

As an infectious disease mostly affecting the lungs, tuberculosis (TB) is caused by the *Mycobacterium tuberculosis* complex (MTBC). TB remains a leading cause of mortality worldwide. Figures from the World Health Organization (WHO) showed that over 100,000 new cases of tuberculosis are reported every year, and over 10,000 individuals die from the disease (1–4). In recent years, efforts to control tuberculosis have faced significant challenges due to the emergence of drug-resistant strains, including extensively drug-resistant tuberculosis (XDR-TB) and pre-XDR-TB (5). Given the high prevalence, mortality rate, and diagnostic challenges of TB and pre-XDR-TB, further research is crucial for the improving timely and early diagnosis of TB (6, 7).

Diagnostic algorithms for tuberculosis typically start with nucleic acid amplification tests (e.g., Xpert MTB/RIF) or sputum-smear microscopy, as both yield results within 1 day. Positive or inconclusive results may require follow-up testing, such as bacterial culture or drug-susceptibility testing, though culture is not used initially due to its longer processing time of 2 to 6 weeks. Currently, bacterial culture remains the gold standard for diagnosing TB; however, it is time-consuming and dependent on high-quality specimen collection (6, 8). In contrast, nucleic acid amplification testing, such as Xpert MTB/RIF and real-time PCR, offers rapid and accurate result but is too costly for widespread use in rural areas (8, 9). Meanwhile, chest X-rays, while sensitive for diagnosing pulmonary TB, exhibit low specificity (10). These diagnostic modalities, with their respective strengths and limitations, must be selected discreetly based on the clinical context within the diagnostic workflow for tuberculosis.

Noticeably, fluorescence *in situ* hybridization (FISH) emerged in 1999 as an accurate and cost-effective method for MTBC detection and has been widely used in recent years (11–13). FISH is a gene-localization, detection, and identification technique that uses fluorescence-labeled probes hybridized with particular nucleic acid sequences. It offers several benefits, including high sensitivity, specificity, safety, and efficiency (14, 15). The probes of FISH include peptide nucleic acid (PNA), oligonucleotide (Oligo), and deoxyribonucleic acid (DNA). Among them, PNA probe shows strong metabolic stability, high affinity, and enormous sequence specificity (16). DNA probes can be multiplied indefinitely, are not easily degraded (compared to RNA) and are generally effective in inhibiting DNA enzyme activity (17). Oligo can persistently recognize the same chromosome in the target species and thus have wide usage (18). However, current studies have yet to provide a clear answer as to which approach is more effective when applied to FISH assays. FISH is primarily used to diagnose genetic aberration routine, infectious diseases caused by acute intracellular bacteria, and biomembrane-related infections (19–21). Some studies showed that FISH was faster and more practical than the traditional method in diagnosing TB in various specimens (8, 12, 22). Nevertheless, some research has demonstrated that when it comes to direct clinical specimens, the possibility of false negative findings needs to be taken into account (23). To date, no meta-analysis has investigated the value of detecting PTB in sputum samples with FISH.

In order to make an appropriate clinical decision in choosing a proper diagnostic approach, a definitive assessment of whether FISH could enhance predictive performance over traditional approaches is necessary. This is especially vital for low and middle-income countries, which have a high prevalence of TB and need to provide TB preventive medication to all household. According to the most recent guidelines by WHO, an accurate diagnosis approach of tuberculosis is recommended for these individuals (24). Nevertheless, there was scant data from such nations in the earlier research.

The assessment of the diagnostic performance of FISH for TB is urgently needed. Given that relevant studies of the clinical value of FISH are limited and controversial, a meta-analysis is conducted here to comprehensively evaluate the clinical efficacy of FISH in diagnosing PTB.

## Methods

### Search strategy

We searched all literature in the Embase, PubMed, Cochrane Library, and Web of Science databases for studies before July 18, 2024, without regard to geographic limitations. The search was performed by searching MeSH terms and EMTREE terms obtained by “FISH” and “tuberculosis” in PubMed and Embase. We searched English databases in English with no restriction on publication time or the site of tuberculosis but we selected studies that included sputum samples in the following steps to avoid omissions. The detailed search strategies for four databases are reported in Supplementary material. The protocol for this review is registered on PROSPERO.^1^

### Filtering and selection of literature

**Figure 1:**
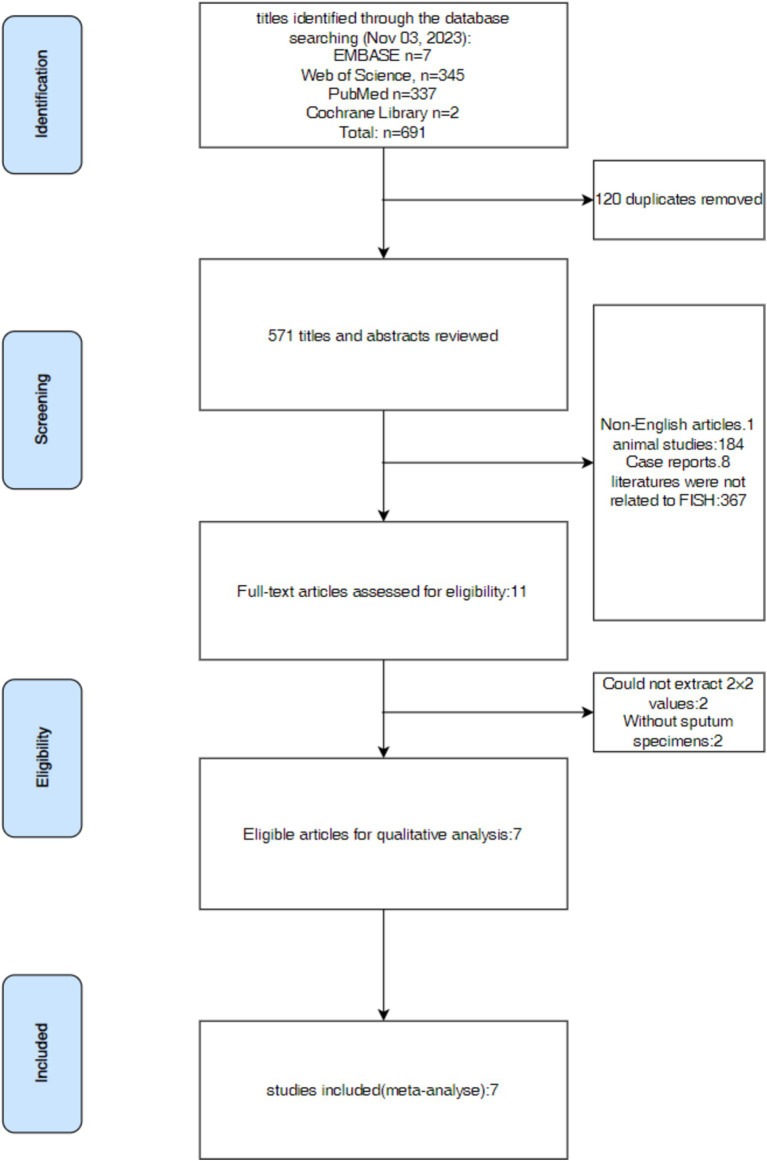
Flow diagram for systematic article search.

The following tasks were independently done by three investigators: Y-QH, KL, and L-QL. They searched pertinent literature, read the abstract and full text of the articles, and extracted data from the 2 × 2 contingency table. The retrieved data was then examined for consistency; if not, the previous procedures were carried out again. The data analysis and figure production were carried out only in cases with integrated data. The disagreements among the three investigators were settled by consulting Y-RH, a fourth investigator. This review procedure was used for all of the reviewed publications in this research. The whole process for selecting was shown in Figure 1.

### Eligibility criteria

Inclusion criteria were as follows: (i) studies that focused on FISH testing using reference standards (such as culture, DNA testing, etc.); (ii) studies that included sputum samples and (iii) studies that offered sufficient information to determine the diagnostic efficacy of FISH. The age or geographic location of the study population did not restrict the included research. Exclusion criteria were as follows: (i) duplicate studies in the literature; (ii) studies using animal models and non-human samples; (iii) abstracts from conferences, lectures, reviews, letters, and case reports; (iv) studies that had incomplete raw data; and (v) publications written in languages other than English.

### Reference method of detection

*Mycobacterium* culture or composite reference criteria (CRS) were utilized as references for detection method in this study. Clinical signs, histology, biochemical test findings, smears, cultures, various nucleic acid amplification assays, and responses to anti-TB medication therapy are involved in the composite reference criteria.

### Statistical analysis and data synthesis

Statistical analyses including the sensitivity (SEN), specificity (SPE), positive likelihood ratio (PLR), negative likelihood ratio (NLR), and diagnostic odds ratio (DOR) with associated 95% confidence intervals (95% CI), were performed using Meta-DiSc software (Ramony Cajal Hospital, Madrid, Spain), (version 1.4). The total accuracy was then assessed using the area under the summary receiver operating characteristic (SROC) curve. Using Stata software, version 12.0 (StataCorp) and version 16.0 (StataCorp LLC, College Station, TX, United States), we constructed univariate-random effects logistic regression models, while studies only provided sensitivity estimates. Fagan’s nomogram and a bivariate boxplot were used to assess the outliers and characterize the FISH diagnostic value for TB. We built a Deeks funnel plot to assess potential publishing bias graphically.

Heterogeneity in meta-analyses represents a significant level of variation in research findings (25). This heterogeneity may result from differences in research quality, test techniques and thresholds between studies (25). Pooled summary estimates from meta-analyses are difficult to evaluate when there is high heterogeneity involved. We used subgroup (stratified) analyses to look at heterogeneity. To explore the accuracy of FISH detection in countries with different TB incidences, references included in our study are classified into subgroups originating from low-TB incidence and middle-and-high-TB incidence. Among them, nations with an annual TB incidence rate of less than 20 cases per 100,000 population are classified as low-incidence nations according to the statistics published by WHO (26). Moreover, we divided studies into three different groups according to specific probes used in the research. We were able to ascertain whether a specific probe or pulmonary tuberculosis incidence was more likely to be linked to improved accuracy using subgroup analysis.

## Results

### Study results & characteristics

We gathered 8 data groups from the 7 articles (Research by Borekci included 2 data groups using Oligo-probe and PNA-probe separately.) including a total number of 1,224 samples that were chosen. We were able to identify data from this research, including author, study design, detection technique, year, country, sample type, and sample source. Among the 8 records, four reported the diagnostic effectiveness of FISH in sputum samples using PNA probe, three explored the diagnostic effectiveness of FISH in sputum samples using Oligo probe, while the last one used DNA probe. All the articles were retrospective, including participants from India, Turkey, Denmark, Korea, America, China, and France. Table 1 provides an overview of the specific features of the included research.

**Table 1:** Characteristics of the included studies.

Author Year	Country	Study type	Detection method	Specimen	Age	Golden standard	Bacterial type	TP	FP	FN	TN	Sensibility	Specificity
Baliga et al. (12)	India	Retrospective study	DNA-FISH	202 clinical sputum specimens	/	DNA sequencing	MTBC	61	6	7	128	89.70%	95.52%
Borekci et al. (22)	Turkey	Retrospective study	Oligo-FISH	44 clinical sputum specimens	/	Acid-fast staining method	MTBC	41	1	1	1	97.62%	50.00%
Borekci et al. (24)	Turkey	Retrospective study	PNA-FISH	44 clinical sputum specimens	/	Acid-fast staining method	MTBC	39	0	4	1	90.70%	100.00%
Stender et al. (31)	Denmark	Retrospective study	PNA-FISH	72 clinical sputum specimens	/	Acid-fast staining method	MTBC	46	0	4	22	92.00%	100.00%
Kim et al. (23)	Korea	Retrospective study	Dual-color PNA-FISH	140 clinical sputum specimens	/	Culture	/	82	0	18	40	82.00%	100.00%
Shah et al. (13)	America	Retrospective study	PNA-FISH	243 clinical sputum specimens	/	DNA sequencing	*M. tuberculosis H37Rv*, *M. tuberculosis RV 37 Ra*, *M. bovis*, *M. bovis*—*BCG*, *M. microti*, *M. africanum*	6	0	0	66	100.00%	100.00%
Yuan et al. (8)	China	Retrospective study	rRNA-specific oligonucleotide probe	542 clinical sputum specimens	>18	Culture	*M. tuberculosis*, *M. avium* subsp. *avium*, *M. avium* subsp. *paratuberculosis*, *M. intracellulare*, *M. kansasii*, *M. abscessus*, *M. phlei*, *M. parafortuitum*, *Bacillus subtilis*, *Escherichia coli*, and *Corynebacterium* sp.	37	7	8	482	82.00%	98.00%
Loukil et al. (35)	France	Retrospective study	Oligonucleotide probe, rpoBMTC-FISH	116 sputum specimens	/	Ziehl–Neelsen staining	*Mycobacterium tuberculosis*	31	0	0	85	100.00%	100.00%

MTBC, *Mycobacterium tuberculosis* complex; TP, true positive; FP, false positive; FN, false negative; TN, true negative; PNA, peptide nucleic acid; Oligo, oligonucleotide, DNA, deoxyribonucleic acid.

### SROC curve and diagnostic accuracy

**Figure 2:**
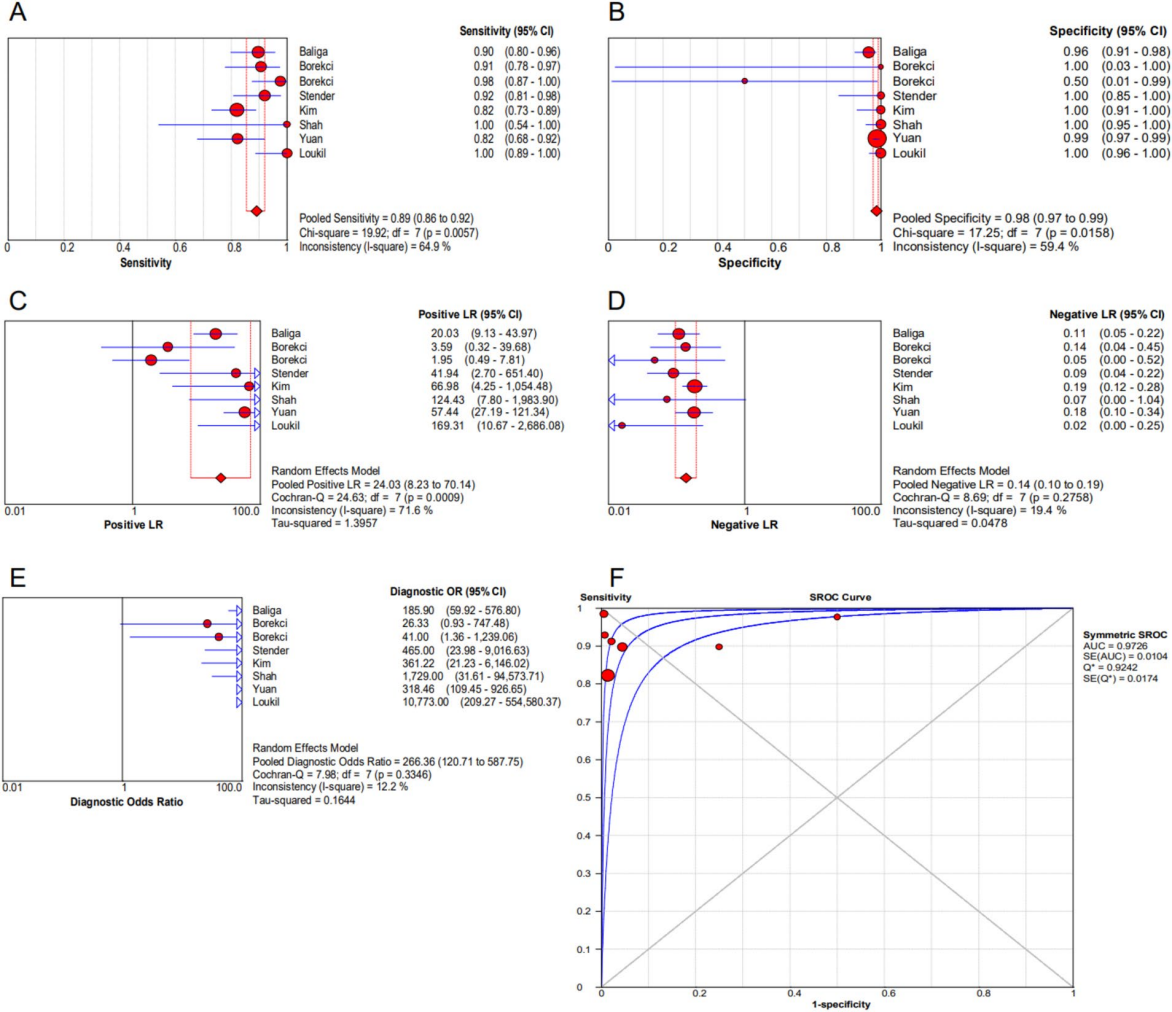
Forest plot of FISH assay. **(A)** Sensitivity. **(B)** Specificity. **(C)** Positive LR. **(D)** Negative LR. **(E)** Diagnostic odds ratio. **(F)** SROC curve.

**Figure 3:**
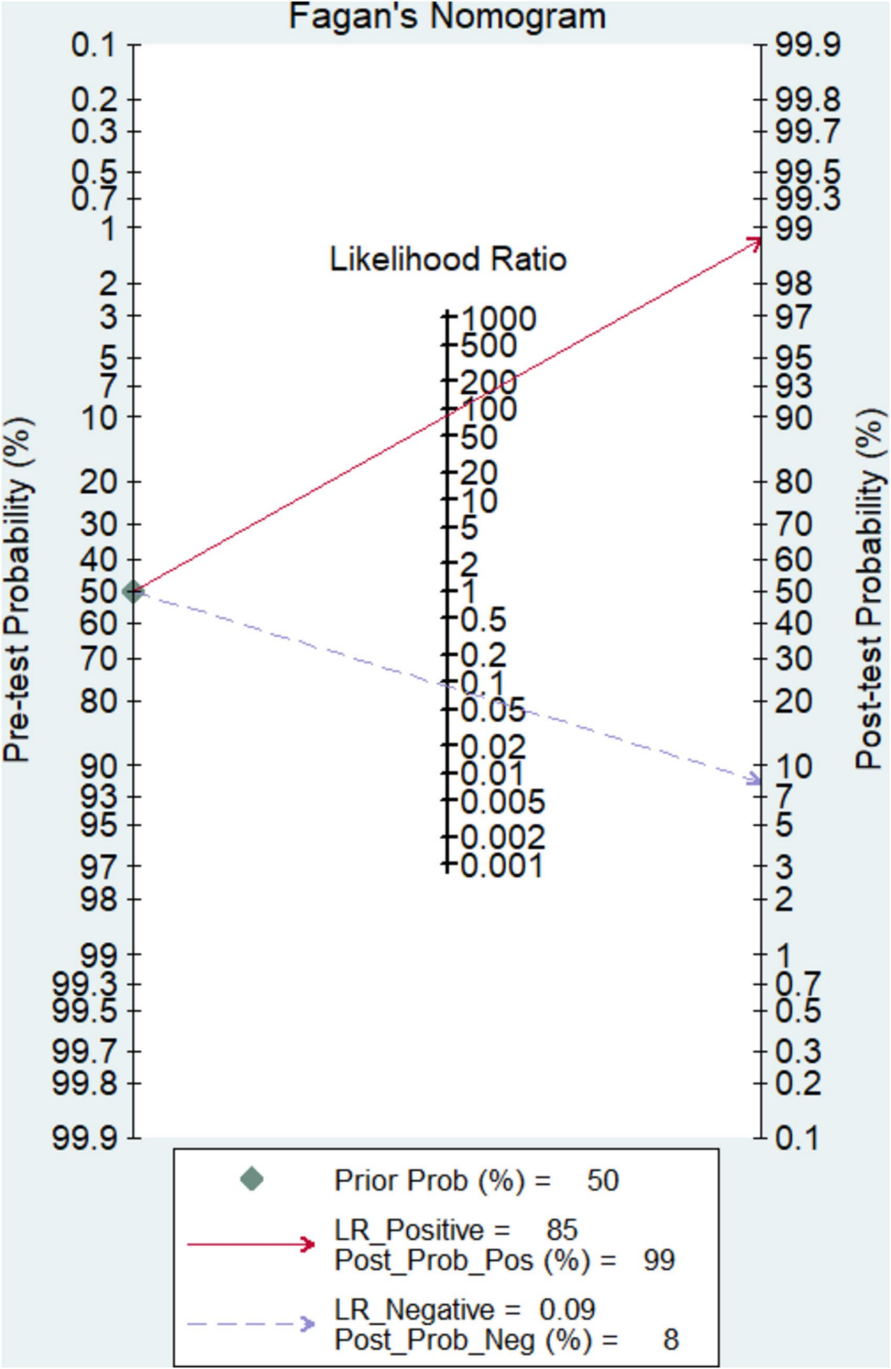
Fagan nomogram analysis of FISH assay.

As can be seen in Figures 2A,B, the overall diagnostic sensitivity and specificity of FISH in detection PTB were 0.89 (95% CI 0.86–0.92) and 0.98 (95% CI 0.97–0.99). The PLR of FISH was 24.03 (95% CI 8.23–70.14, Figure 2C), and the NLR was 0.14 (95% CI 0.10–0.19, Figure 2D). As shown in Figure 2E, the DOR result was 266.36 (95% CI 120.71–587.75). FISH had an AUC (area under the SROC curve) of 0.9726 and a Q^*^ index of 0.9242 (Figure 2F). Based on a 50% anticipated chance of positive test results, the Fagan nomogram analysis revealed a 99% positive post-test probability and an 8% negative post-test probability (Figure 3).

### Assessment of methodological quality

The Quality Assessment of Diagnostic Accuracy Studies (QUADAS-2) guidelines, covering four main components—case selection, the trial to be evaluated, the reference standard, and case process and progress, were used by three investigators (Y-QH, KL, and L-QL) to independently assess the quality of the included studies, the results of which were placed in Table 2.

**Table 2:** The quality evaluation results for each study included in the meta-analysis.

Study	Year	QUADAS-2										
		1	2	3	4	5	6	7	8	9	10	11
Baliga	2018	Y	Y	Y	N	Y	Y	Y	Y	Y	Y	Y
Börekçi	2014	Y	Y	Y	N	Y	Y	Y	Y	Y	Y	Y
Stender	1999	Y	Y	Y	N	Y	Y	Y	Y	Y	Y	Y
Romero	2005	Y	Y	Y	Y	Y	Y	N	Y	Y	Y	Y
Rodriguez-Nuñez	2012	Y	N	Y	N	Y	Y	Y	Y	Y	Y	Y
Kim	2015	Y	N	Y	N	Y	Y	Y	Y	Y	Y	Y
Shah	2017	UC	Y	Y	Y	Y	Y	Y	Y	Y	Y	N
Yuan	2015	Y	Y	Y	Y	Y	Y	Y	Y	Y	Y	N
Lefmann	2006	UC	N	Y	Y	Y	Y	N	Y	Y	Y	Y
Loukil	2018	Y	N	Y	N	N	Y	Y	Y	Y	Y	Y

N, no; UC, unclear; Y, yes.

### Methodological quality evaluation

**Figure 4:**
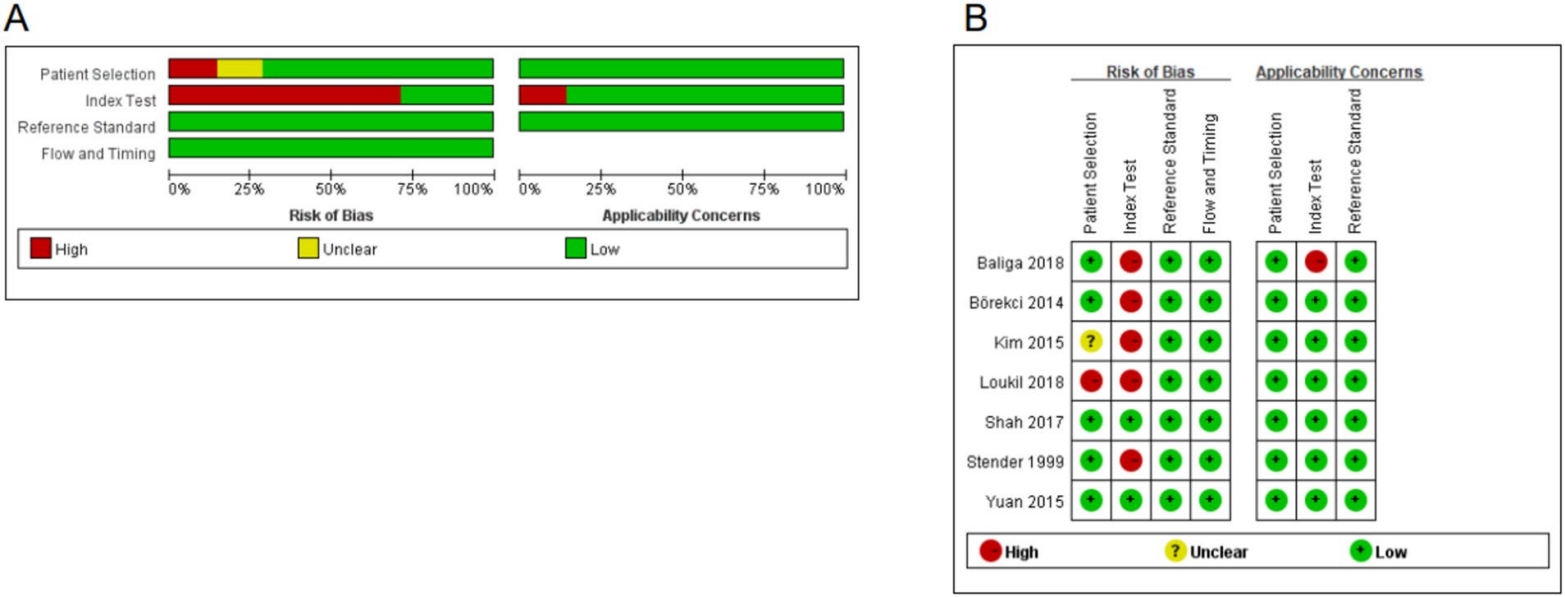
Quality assessment of the included studies. **(A)** Overall quality assessment of included studies. **(B)** Quality assessment of the individual studies.

Review Manager 5.3 was used to examine the overall quality (Figure 4B) and the quality of individual studies (Figure 4A). The findings demonstrated that: (i) for the patient section criterion, one study had a high risk of bias. (ii) Index test was high in five studies in which test assessors were not masked to tuberculosis gold standard results such as culture or sequencing. (iii) Studies included were all considered to be at low risk of bias in the field of “flow and timing” and “reference standard.”

### Heterogeneity analysis

**Figure 5:**
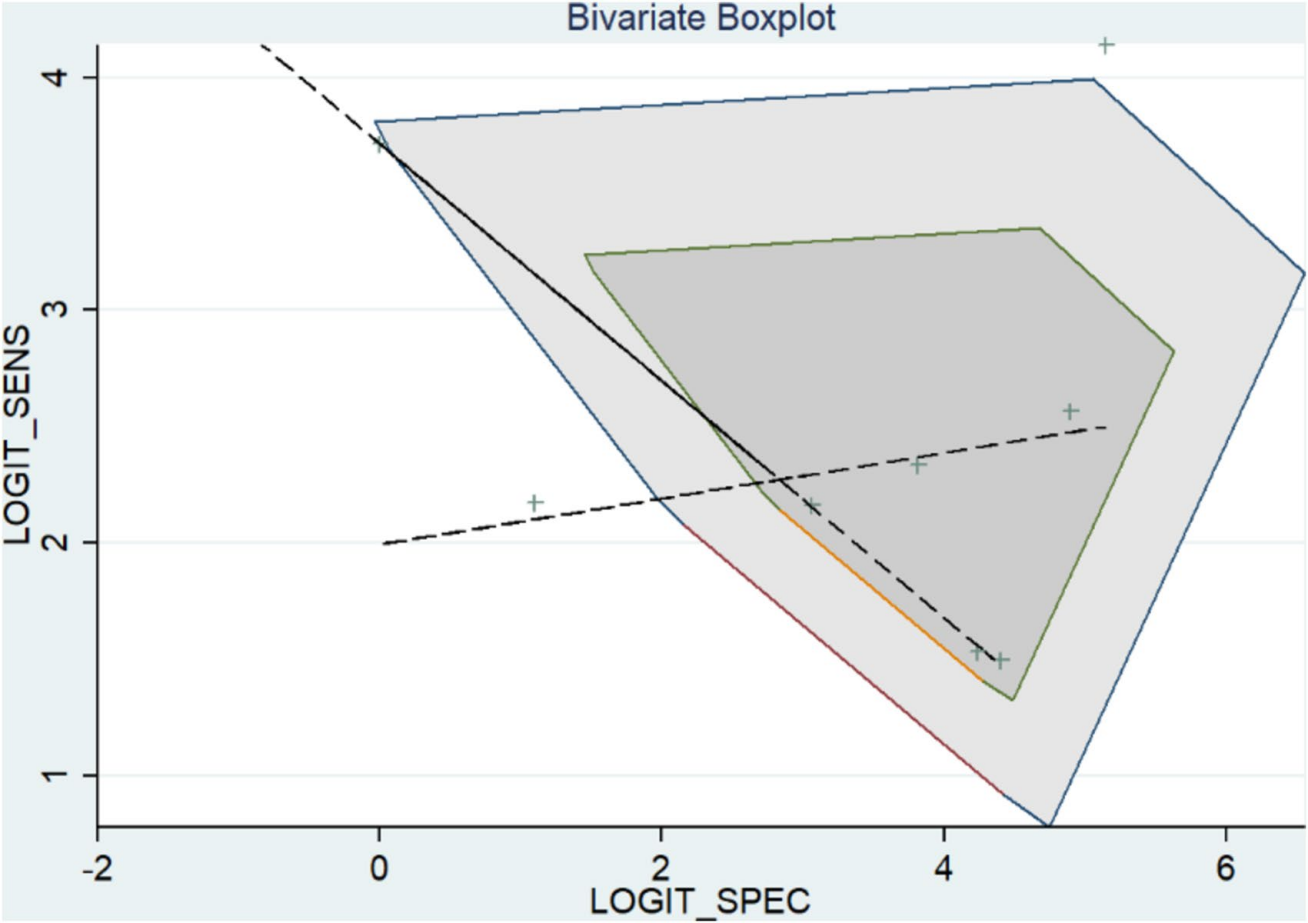
Bivariate boxplot of FISH assay.

To examine the heterogeneity, we used the *bivariate boxplot* and index (I-square). Three of the data sets for FISH were outside of the circles, according to the *bivariate boxplot* (Figure 5).

### Subgroup meta-analyses

Subgroup analyses were performed to analyze the performances of various probe usage and TB incidence of the collected data. In studies using PNA to identify PTB, the specificity of PNA-FISH was at 100% (*p* = 1.0000, *I*^2^ = 0.0%) and sensitivity was at 87% (*p* = 0.1384, *I*^2^ = 45.5%). In studies using Oligo to identify PTB, the specificity of Oligo-FISH was at 99% (*p* = 0.0166; *I*^2^ = 75.6%) and sensitivity was at 92% (*p* = 0.0024, *I*^2^ = 83.4%). FISH’s specificity was 100% (*p* = 1.0000, *I*^2^ = 0.0%) and its sensitivity was 95% (*p* = 0.1016, *I*^2^ = 56.3%) in nations with low TB incidence. Among middle and high TB incidence countries, the specificity of FISH was at 98% (*p* = 0.0299, *I*^2^ = 62.7%) and sensitivity was at 87% (*p* = 0.0420, *I*^2^ = 59.6%). The diagnostic odds ratio (DOR) among low TB incidence countries was at 1516.05 (*p* = 0.4451, *I*^2^ = 0.0%) while at 211.23 (*p* = 0.5352, *I*^2^ = 0.0%) among middle and high TB incidence countries (Table 3).

**Table 3:** Included data by subgroups.

Covariate	Subgroup	Meta-analytic summary estimates					
		Sensitivity, % (95% CI)	*p*-value	Specificity, % (95% CI)	*p*-value	Diagnostic OR (95% CI)	*p*-value
Probes	PNA	87 (0.81–0.91)	0.1384	100 (0.97–1.00)	1.0000	274.27 (55.31–1360.09)	0.4121
	Oligo	92 (0.86–0.96)	0.0024	99 (0.97–0.99)	0.0166	408.40 (36.68–4547.47)	0.0932
TB incidence	Low TB-incidence country	95 (0.89–0.99)	0.1016	100 (0.98–1.00)	1.0000	1516.05 (197.38–11644.72)	0.4451
	Middle and high TB-incidence country	87 (0.83–0.91)	0.0420	98 (0.96–0.99)	0.0299	211.23 (103.34–431.76)	0.5352

OR, odds ratio; CI, confidence interval; TP, true positive; FP, false positive; FN, false negative; TN, true negative; PNA, peptide nucleic acid; Oligo, oligonucleotide; DNA, deoxyribonucleic acid.

### Publications bias evaluation

**Figure 6:**
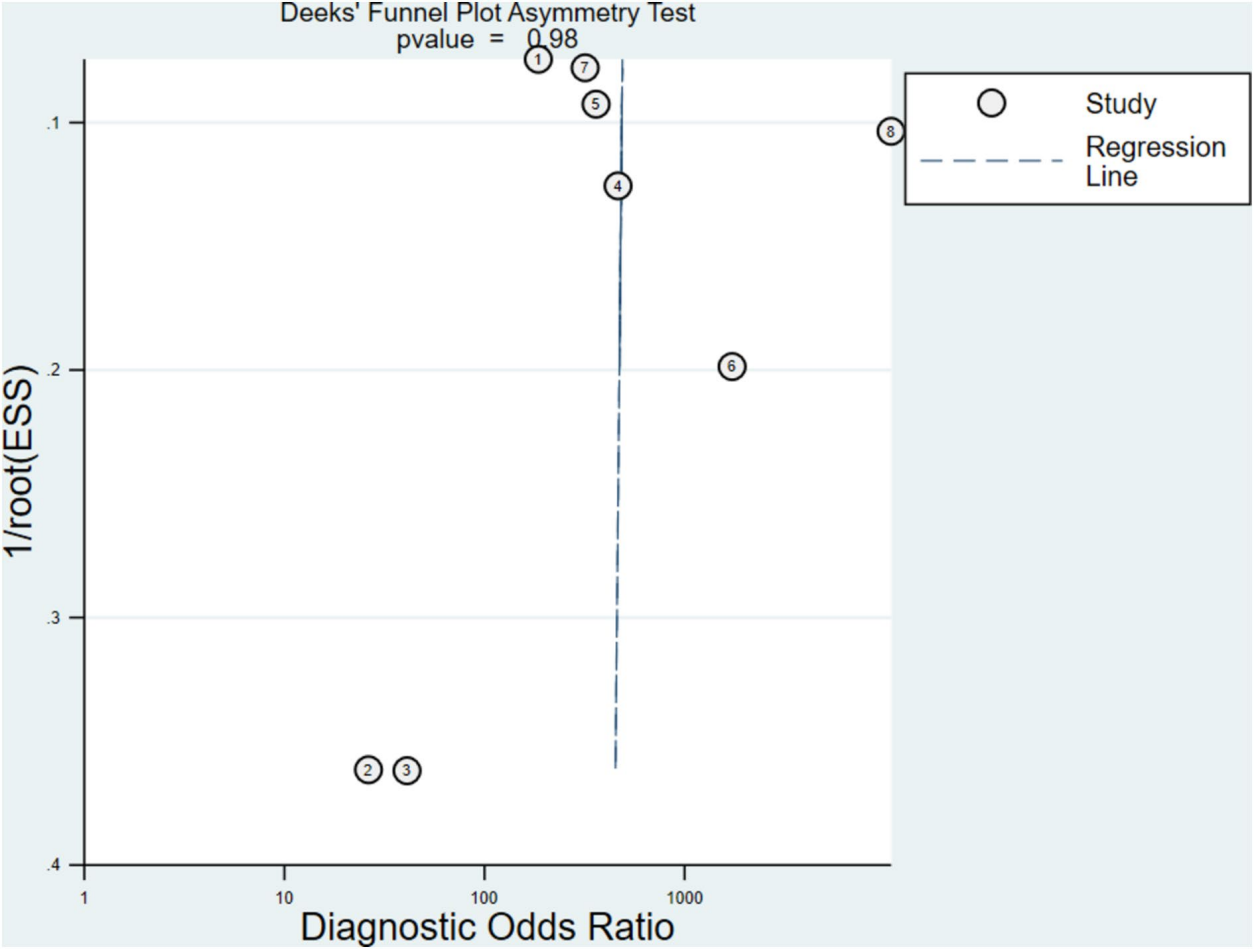
Deeks’ funnel plot asymmetry test of FISH assay.

Compared to non-statistically significant research, statistically significant results have a higher chance of being accepted and published in comparable investigations. Publication bias is hard to manage and affects how systematic evaluations turn out. Figure 6 displays the Deeks’ funnel plot with a *p*-value of 0.98 for the FISH experiment which implies insignificant bias.

## Discussion

This systematic review and meta-analysis concluded that the likelihood of diagnosing tuberculosis was enhanced when MTBC was found in sputum samples using FISH as compared to standard bacterial culture. FISH has been shown to be a fast and highly reliable technique with high sensitivity (12).

In our study, we found that FISH assays showed a sensitivity of 0.89 (95% CI 0.86–0.92), a specificity of 0.98 (95% CI 0.97–0.99), and a DOR of 266.36 (95% CI 120.71–587.75). The high AUC (0.9726) and *Q* index (0.9294) of the SROC curve for FISH, indicated that FISH assays have a notably high value for the diagnosis of TB. The high sensitivity may be attributed to its ability to target thousands of copies of rRNA in the bacteria, facilitating direct visualization of bacterial distribution and morphology. The high specificity may be due to the specific probe design (27). It can reliably differentiate between MTBC and non-tuberculous mycobacteria (NTMs).

Stratification analysis by national TB incidence revealed significant differences in DOR between countries with high and low prevalence of TB, with superior predictive performance observed in low-prevalence settings (211.23 for high-prevalence countries and 1516.05 for low-prevalence countries). The observed heterogeneity in test performance aligns with findings from other diagnostic methods (28) and may arise from environmental factors such as routine BCG vaccination at birth, high exposure rates to tuberculosis, co-infection with HIV, and frequent contact with NTMs or *helminth* infections in high TB burden areas. Additionally, the likelihood of contracting TB before testing is higher in high-prevalence nations, which reduces the negative predictive value (NPV), and the sensitivity is decreased since individuals who test negative at baseline have a higher chance of contracting TB (29). Another possible reason is the high mutation rate of bacteria in high-prevalence countries, due to the different transmission dynamics of tuberculosis and the increased number of breeding generations, leading to a decrease in diagnostic performance. Thus, in countries with a high prevalence of TB, tests with higher sensitivity, such as biomarkers in the blood (30), are still needed to predict and diagnose the disease. Furthermore, special populations like HIV-infected patients, household with individuals infected with tuberculosis, and those receiving immunotherapy can be treated prophylactically, as recommended by the WHO (24), with latent tuberculosis infection (LTBI) testing or direct prophylaxis, which will reduce the burden for detection and diagnosis in countries with high prevalence.

Since 1999, there have been numerous studies using FISH to detect MTBC, and most of them have employed PNA-FISH, which is a quick and precise method for identifying culture-grown mycobacteria by species (31). Another method called Oligo-FISH is useful for studying allopolyploid identification, chromosome variation detection, and interpreting three-dimensional (3D) genomic structures (32, 33). Because of the scarcity of researches focusing on the use of DNA-FISH to monitor *Mycobacterium avium*, our subgroup analyses were performed only for Oligo probes and PNA probes. The results of our study showed that PNA-FISH has a higher specificity (100% vs. 99%) but a lower sensitivity (87% vs. 92%) compared with Oligo-FISH, indicating their different usage and great potential in the quick screening of TB. The different performance between Oligo and PNA probes is partially due to sequence similarity with samples and dye bias.

Furthermore, potential sources of heterogeneity in the inclusion studies identified by FISH were examined. Given the documented variation in sensitivity and specificity estimations in previous meta-analyses, the moderate heterogeneity seen within and across studies concerning CD4 cell count, the presence of TB symptoms, and clinical context is not surprising. Secondly, cut-off values of FISH were not clearly presented in most studies but varied by factors such as history of BCG vaccination, HIV infection, or other immunosuppression, which might influence test agreement, especially with the FISH test. Besides, methodological and reference standard differences might also contribute to heterogeneities in the study. Overall, the heterogeneity and underlying differences between studies highlight the variations that existed in the interpretation of WHO guidelines in different clinical settings.

This study included broad inclusion criteria which minimize selection bias and potentially obtain more generalized conclusions. Besides, the pairwise meta-analysis of subgroups was performed to correct for heterogeneity and generate more reliable results. The lack of significant small-study bias, as demonstrated by the funnel-plot analysis could also help to improve the strength of the present study. However, several limitations of this study should be acknowledged. Lack of high-quality research is one of the major limitations. The different members of MTBC detected in the studies, and doubts regarding the validity of network meta-analysis are some of the limitations (34). Moreover, if the diagnostic assay is primarily tested on patients with milder symptoms or with a more homogeneous disease profile, the resulting data on sensitivity and specificity may appear more favorable than the actual situation, a phenomenon known as “spectrum bias.” Therefore, comprehensive analysis combined with stricter methods are necessary in the future to determine the real impact of FISH approaches in clinical practice.

As a microscopic and non-invasive approach, FISH offers great potential to provide information for the detection of important pathogens in sputum samples and spatial resolution. Another key significance of this systematic review and network meta-analysis is to guide countries with high and low prevalence of TB to use FISH for more accurate diagnosis of TB. Those countries with a high prevalence of TB still need diagnosis method with higher sensitivity.

## Data Availability

The original contributions presented in the study are included in the article/Supplementary material, further inquiries can be directed to the corresponding author.

## Footnotes

^1^ https://www.crd.york.ac.uk/PROSPERO/display_record.php?RecordID=499771
