# Supplementary material for: Evaluation of the diagnostic efficiency of fluorescence in situ hybridization for pulmonary tuberculosis: a systematic review and meta-analysis
Source: Front Med (Lausanne). 2025 Jan 6;11:1467530. doi: 10.3389/fmed.2024.1467530 (PMC11742950; doi:10.3389/fmed.2024.1467530)
Supplement: Supplementary file 1 [file Table_1.DOCX]

Search strategy for FISH detecting TB based on PubMed, EMBASE, Web of Science and Cochrane Library

| Database | Search Strategy | Results |
| --- | --- | --- |
| PubMed | ((((((((((((FISH assays) OR (FISH study)) OR (Fluorescence in situ hybridization (FISH) Test)) OR (fluorescence in situ hybridization with DNA probes)) OR (A DNA probe-based FISH assay)) OR (A PNA-based FISH assay)) OR (Dual color fluorescence in situ hybridization (FISH) assays)) OR (MTBC FISH assay)) OR (Dual-color PNA FISH)) OR (PNA-FISH methods)) OR (Oligo-FISH methods)) OR (peptide nucleic acid-fluorescence in situ hybridization)) AND (((Mycobacterium tuberculosis) OR (MTB)) OR (Mycobacterium tuberculosis H37Rv)) | 337 |
| EMBASE | #1: 'bacillus tuberculosis'/exp OR 'bacillus tuberculosis' OR 'bacterium tuberculosis' OR 'human tubercle bacillus' OR 'koch bacillus' OR 'koch`s bacillus' OR 'mycobacteria tuberculosis' OR 'mycobacterium tuberculosin' OR 'mycobacterium tuberculosis cultivation' OR 'mycobacterium tuberculosis hominis' OR 'mycobacterium tuberculosis isolation' OR 'mycobacterium tuberculosum' OR 'tubercle bacilli' OR 'tubercle bacillus' OR 'tuberculosis hominis, mycobacterium' OR 'tuberculosis, mycobacterium'  #2: 'fish assays' OR 'fish study' OR 'fluorescence in situ hybridization (fish) test' OR 'fluorescence in situ hybridization with dna probes' OR 'a dna probe-based fish assay' OR 'a pna-based fish assay' OR 'dual color fluorescence in situ hybridization (fish) assays' OR 'mtbc fish assay' OR 'dual-color pna fish' OR 'pna-fish methods' OR 'oligo-fish methods' OR 'peptide nucleic acid-fluorescence in situ hybridization'  #1 AND #2 | 7 |
| Web of Science | #1: ((ALL=(Mycobacterium tuberculosis)) OR ALL=(MTB)) OR ALL=(Mycobacterium tuberculosis H37Rv)  #2: (((((((((((ALL=(FISH assays)) OR ALL=(FISH study)) OR ALL=(Fluorescence in situ hybridization (FISH) Test)) OR ALL=(fluorescence in situ hybridization with DNA probes)) OR ALL=(A DNA probe-based FISH assay)) OR ALL=(A PNA-based FISH assay)) OR ALL=(Dual color fluorescence in situ hybridization (FISH) assays)) OR ALL=(MTBC FISH assay)) OR ALL=(Dual-color PNA FISH)) OR ALL=(PNA-FISH methods)) OR ALL=(Oligo-FISH methods))OR ALL=(peptide nucleic acid-fluorescence in situ hybridization))  #1 AND #2 | 345 |
| Cochrane Library | #1 Mycobacterium tuberculosis 1399  #2 MTB 635  #3 Mycobacterium tuberculosis H37Rv 8  #4 #1 OR #2 OR #3 1781  #5 FISH assays 93  #6 FISH study 6532  #7 Fluorescence in situ hybridization (FISH) Test 116  #8 fluorescence in situ hybridization with DNA probes 25  #9 A DNA probe-based FISH assay 0  #10 A PNA-based FISH assay 0  #11 Dual color fluorescence in situ hybridization (FISH) assays 0  #12 MTBC FISH assay 0  #13 Dual-color PNA FISH 0  #14 PNA-FISH methods 2  #15 Oligo-FISH methods 0  #16 peptide nucleic acid-fluorescence in situ hybridization 1  #17 #5 OR #6 OR #7 OR #8 OR #9 OR #10 OR #11 OR #12 OR #13 OR #14 OR #15 OR #16 6560  #18 #4 AND #17 2 | 2 |
